# Predictive association between control measures and chikungunya fever incidence based on random forest and SHAP analysis

**DOI:** 10.1371/journal.pntd.0014675

**Published:** 2026-09-03

**Authors:** Fengling Chen, Jinsen He, Huihui Liu, Zhilin Wu, Jiwen Wu, Kunlu He, Hongjuan Wen, Xiaohua Li, Hongwei Wu, Lang Lang, Lijie Zhang

**Affiliations:** 1 Chancheng District Center for Disease Control and Prevention, Foshan, Guangdong, China; 2 Chinese Field Epidemiology Training Program, Chinese Center for Disease Control and Prevention (Chinese Academy of Preventive Medicine), Beijing, China; 3 Shunyi District Center for Disease Control and Prevention, Beijing, China; 4 The Johns Hopkins University, Baltimore, Maryland, United States of America; Colorado State University, UNITED STATES OF AMERICA

## Abstract

**Background:**

Using the 2025 Chikungunya fever (CHIK) outbreak in Chancheng District, Foshan City, this study applied a random forest (RF) regression model combined with SHapley Additive exPlanations (SHAP) to explore predictive associations, nonlinear relationships and potential thresholds between environmental–social factors and village/community-level cumulative incidence, to inform stratified control of mosquito-borne diseases.

**Methodology/principal findings:**

In this cross-sectional ecological study of 143 villages/communities, the outcome was cumulative incidence, and nine candidate covariates were assessed, including the hospitalization isolation rate, construction-site density, and population density. Multicollinearity was checked using the variance inflation factor. Model fit was evaluated by the out-of-bag (OOB) R², RMSE, and MAE, and variable importance by %IncMSE. Robustness was tested with 100 repeated runs, bootstrap thresholds from SHAP dependence plots, and a sensitivity analysis excluding the endogenous isolation rate. On the log(1 + incidence) scale, the OOB R² was 0.206, with underestimation of high-incidence areas. The hospitalization isolation rate had the highest importance (%IncMSE = 18.43) and was negatively correlated with predictions (ρ = −0.749), but this likely reflects reverse causation and is predictive only. Construction-site density was strongly positive (ρ = 0.855), with a stable threshold near 12.8 sites/km²; it remained the most robust predictor after removing the isolation rate (%IncMSE = 8.54).

**Conclusions/significance:**

Construction-site density was the most robust environmental predictor, whereas population density contributed little. These exploratory, predictive associations—not causal effects—should guide risk stratification and require prospective validation with time-matched longitudinal data.

## Author summary

Chikungunya fever is a mosquito-borne illness causing fever and prolonged joint pain. In recent years, outbreaks have grown in scale worldwide, posing serious public health challenges. In 2025, Foshan City in southern China experienced the largest locally acquired chikungunya outbreak ever recorded in the country. We focused on Chancheng District, the urban core of Foshan, where 1404 cases were reported across 143 villages and communities. Our question was: why did some communities have far more cases than others, even within the same district. Using an interpretable machine-learning approach (random forest with SHAP analysis), we examined which environmental and social factors predicted community-level incidence. The density of small construction sites—which readily accumulate discarded water-holding containers where mosquitoes breed—was the most stable environmental predictor: risk climbed sharply only after crossing a clear threshold (about 13 sites per square kilometer), rather than rising gradually. By contrast, overall population density had little predictive value in this already crowded setting, suggesting that fine-scale environmental exposure matters more than population size. The hospitalization isolation rate showed the strongest statistical association with lower incidence, but we interpret it cautiously: communities that brought their outbreaks under control tended to reach higher isolation rates, so this likely reflects the response to the epidemic rather than proof that isolation reduced transmission. As an exploratory analysis based on data from a single time point, our study points to predictive associations useful for flagging high-risk communities and targeting limited control resources—not causal effects—and prospective studies are needed to confirm them.

## 1. Introduction

Chikungunya fever (CHIK) is an acute infectious disease caused by the chikungunya virus (CHIKV), primarily transmitted by Aedes mosquitoes. Recent global outbreaks of CHIK have posed significant public health challenges [1–4]. CHIKV shares its main vectors, Aedes aegypti and Aedes albopictus, with dengue and Zika viruses and often co-circulates with these pathogens in endemic regions. Additionally, CHIK outbreaks exhibit cyclical patterns, typically occurring at inter-epidemic intervals of 7–8 years, complicating both differential diagnosis and disease control efforts [5–9]. Since China’s first imported CHIKV case was reported in Guangdong Province in 2008 [10], the majority of subsequent cases have been sporadic and imported, without establishing sustained local transmission [11].

In July 2025, Foshan City in Guangdong Province experienced the largest documented local CHIK cluster outbreak in China. By the outbreak’s conclusion, approximately 25,517 laboratory-confirmed cases were reported across 21 prefecture-level cities in Guangdong Province, of which 10,847 cases occurred in Foshan City alone. Chancheng District, the central urban district of Foshan City, reported 1,404 cases, accounting for 12.94% (1,404/10,847) of the city’s total cases. Prior studies estimated the basic reproduction number (R_0_) for this outbreak to be as high as 16.3 (95% CI: 15.0–17.5), with a doubling time of just 3.5 days [12], far exceeding the magnitude of the 2010 Dongguan outbreak (R_0_ = 5.5; 253 cases) [13]. Through comprehensive interventions targeting both infection-source management and vector control, Chancheng District successfully contained the outbreak by November 2025.

Current methods to evaluate mosquito-borne disease control measures are typically categorized as mechanistic models based on transmission dynamics or statistical and machine-learning models that rely on historical data [14]; however, both approaches have notable limitations [15]. Mechanistic models (e.g., SEIR models) simulate theoretical disease transmission dynamics but depend heavily on accurate prior assumptions about transmission biology. Their results are highly sensitive to parameters such as transmission rates and R_0_, which are difficult to accurately estimate and vary dynamically in real-world scenarios, limiting their empirical utility [16–23]. Conventional statistical models (e.g., linear regression, logistic regression) are commonly used to identify risk factors but typically assume linear relationships between predictors and outcomes, making them unsuitable for capturing complex nonlinear interactions often encountered in real-world settings [24,25]. Consequently, these models may inadequately assess the combined effects of multiple interventions. Machine-learning models have demonstrated improved performance in outbreak prediction [26,27]; however, prior studies have mainly focused on case forecasting [14,15,18,28,29], with fewer applications in the retrospective evaluation of control measures. To address these methodological limitations, the present study employs random forest (RF) models for predictive assessment [26]. RF approaches require no prior mechanistic assumptions, leverage multi-source surveillance data directly, naturally capture complex nonlinear relationships and variable interactions, and eliminate the need for pre-specified functional forms. Additionally, RFs provide built-in measures of variable importance, facilitating the identification of key determinants [25,30]. This methodological innovation offers a robust and nuanced approach to characterizing predictive associations involving control measures, addressing an important research gap characterized by an emphasis on prediction rather than evaluation, and expanding the use of advanced data-mining techniques for predictive assessment.

Using data from the 143 villages/communities in Chancheng District, this study applied RF regression modeling combined with SHAP analysis to explore predictive relationships and nonlinear patterns linking environmental and social factors, such as hospitalization isolation rate, construction-site density, and population density, to cumulative village/community-level CHIK incidence. The study aimed to provide evidence-based insights for developing targeted prevention and control strategies against future mosquito-borne disease outbreaks.

## 2. Materials and methods

### 2.1. Ethics approval and consent to participate

Ethical review was not required for this study because the analysis was based on anonymized, publicly available surveillance data and did not involve medical intervention, biological sample collection, or any impact on patient clinical management.

### 2.2. Study design

This research employed a cross-sectional ecological design with villages/communities as analysis units. Aggregated data from the 2025 CHIK outbreak in Chancheng District, Foshan City, were analyzed to identify associations between village/community-level environmental and social characteristics and cumulative disease incidence. Findings reflect group-level associations and do not represent individual-level relationships.

### 2.3. Data sources

Outbreak data, comprising all laboratory-confirmed CHIK cases in Chancheng District during 2025, were retrieved from the China Information System for Disease Control and Prevention. Village/community-level population data were obtained from Chancheng District Public Security Bureau statistics as of year-end 2024. Data concerning administrative areas and environmental features, including construction sites, narrow alleys (locally termed “cold lanes”), and vacant houses, were provided by each town/subdistrict.

### 2.4. Variable definitions

#### 2.4.1. Dependent variable.

The dependent variable was the cumulative incidence of CHIK per village/community (‰), calculated as follows: cumulative incidence = (total reported cases/resident population) × 1000‰.

#### 2.4.2. Independent variables.

A total of nine candidate village/community-level covariates were compiled, of which eight were included in the main random forest model; the Wolbachia-based intervention (abbreviated “mosquitoes against mosquitoes”) was analyzed descriptively only:

**① Hospitalization isolation rate (hosp_rate):** the percentage of reported cases admitted to medical institutions for mosquito-proof isolation and treatment, calculated as (number of hospitalized isolated cases/total number of reported cases) × 100%.

**② Population density (pop_den):** resident population of each village/community/administrative area (10,000 persons/km²).

**③ Green-space coverage (green_rate):** proportion (%) of self-managed greenery area within each village/community, including small parks and vegetable plots but excluding larger parks managed at or above town/subdistrict level and greening strips along public roads (area of self-managed greenery/administrative area × 100%).

**④ Construction-site density (const_den):** number of self-managed construction sites per administrative area of each village/community (sites/km²). These sites include small-scale projects such as elevator retrofits, self-built rural houses, and urban-village housing sites, but exclude district-level construction projects.

⑤ **Narrow-alley density (alley_den):** number of narrow alleys (locally termed “cold lanes”) per unit administrative area (alleys/km²). A “cold lane” refers to a narrow, longitudinal passage within traditional Lingnan-style architecture or narrow open-air alleys between adjacent buildings. Such lanes typically exhibit poor ventilation, high humidity, and standing-water containers, creating suitable breeding and resting habitats for Aedes mosquitoes.

⑥ **Vacant-house density (idle_den):** number of vacant houses per unit administrative area within each village/community (houses/km²).

⑦ **Rooftop density (roof_den):** number of rooftops in each village/community/administrative area (rooftops/km²).

⑧ **Outdoor mosquito-killing lamp density (lamp_den):** number of ultraviolet or solar-powered outdoor mosquito-killing lamps within each village/community/administrative area (lamps/km²).

⑨ **Wolbachia-based mosquito control (“mosquitoes against mosquitoes,” biocontr):** between 29 April and December 2025, five villages/communities representing the east, south, west, north, and central areas of Chancheng District were selected for weekly releases of 15,000–75,000 Wolbachia-infected sterile male mosquitoes. This strategy aimed to suppress wild mosquito populations and reduce vector-borne transmission risk.

Because the “mosquitoes against mosquitoes” measure covered only 5 villages/communities, accounting for 3.50% (5/143), the small sample size did not meet the modeling requirements; therefore this indicator was analyzed by descriptive statistics only and was not included in the core random forest model.

***Note on variable timing:*** *Environmental variables were measured in August 2025 and were assumed to be relatively stable over the short term. However, the hospitalization isolation rate was time-dependent and may have changed in response to outbreak intensity and control activities.*

### 2.5. Statistical methods

#### 2.5.1. Analysis software and environment.

All statistical analyses in this study were performed in R 4.5.2. Random forest models were built using the randomForest package; SHAP values were calculated using the fastshap package and visualized using the shapviz package; data cleaning, processing, and plotting were performed using packages such as dplyr, tidyr, tibble, ggplot2, and patchwork.

#### 2.5.2. Random forest model construction.

The village/community cumulative incidence was used as the outcome variable and was log-transformed as log(1 + cumulative incidence) before modeling. The main model included eight village/community-level covariates: population density, construction-site density, vacant-house density, rooftop density, narrow-alley density, green-space coverage, outdoor mosquito-killing lamp density, and hospitalization isolation rate. The Wolbachia-based intervention covered only 5 villages/communities; because the sample size was too small, it was not included in the model and was analyzed by descriptive statistics only. The percentage increase in mean squared error (%IncMSE) was used to assess the predictive importance of each variable [31].

#### 2.5.3. Model performance evaluation.

The random forest (RF) is a bagging-based ensemble learning method that can be used for both classification and regression problems. Given an original data sample of size n, bootstrap sampling with replacement is used to draw b bootstrap sample sets, from which b regression trees are constructed; the samples not drawn in each bootstrap draw constitute the out-of-bag (OOB) data [32].

Model performance was evaluated based on the OOB prediction results by calculating the out-of-bag coefficient of determination (OOB R²), root mean squared error (RMSE), and mean absolute error (MAE) on both the log-transformed scale and the original incidence scale. Scatter plots of observed versus predicted values and residual-versus-predicted plots were drawn to analyze the distribution of residuals, and groups were divided by quartiles of incidence level to explore the pattern of residual distribution, in order to comprehensively evaluate the overall predictive performance of the model and determine whether the prediction errors were concentrated in high-incidence villages/communities.

#### 2.5.4. Result stability and multicollinearity testing.

To assess the stability of the variable-importance results, this study repeatedly constructed the random forest model 100 times while keeping the model structure, included variables, outcome-variable transformation, and random forest parameters unchanged, altering only the random seed. The mean %IncMSE of each variable, the empirical 2.5%–97.5% percentile range, the mean rank, and the frequency of entering the top three were summarized. The Spearman correlation coefficient combined with the variance inflation factor (VIF) was used to comprehensively test for multicollinearity among the covariates; when the VIF of all variables was < 5 and the absolute value of the pairwise Spearman correlation coefficients did not reach 0.70, it was considered that no significant multicollinearity was present.

#### 2.5.5. SHAP effect analysis and threshold-feature analysis.

The SHAP method was used to interpret the contribution of each predictor to the random forest model’s predictions [33]. The mean absolute SHAP value was used to reflect the overall contribution strength of each variable to the model’s predictions; the Spearman correlation coefficient between each variable’s values and its corresponding SHAP values was used to summarize the direction of the variable’s contribution.

Furthermore, SHAP dependence plots were used to explore the nonlinear relationships between variables and the model’s predicted values; within the 5th–95th percentile range of each variable, the intersection between the LOESS-smoothed SHAP curve and the SHAP = 0 reference line was defined as the “SHAP-derived threshold-like intersection.” The bootstrap method was used to assess the stability of these threshold-like intersections. This analysis was used to describe nonlinear features in the model’s predictions and was not used to infer definitive causal thresholds.

#### 2.5.6. Sensitivity analysis.

The hospitalization isolation rate may simultaneously reflect the severity of the epidemic and the intensity of the local control response, and thus carries a potential risk of endogeneity. To assess the influence of this variable on the main-model results, this study constructed a seven-covariate model excluding this variable for sensitivity analysis. Throughout the sensitivity analysis, the same variable-transformation approach, random forest parameters, out-of-bag evaluation metrics, and 100-run stability analysis were used.

## 3. Results

### 3.1. Outbreak overview

**Fig 1:**
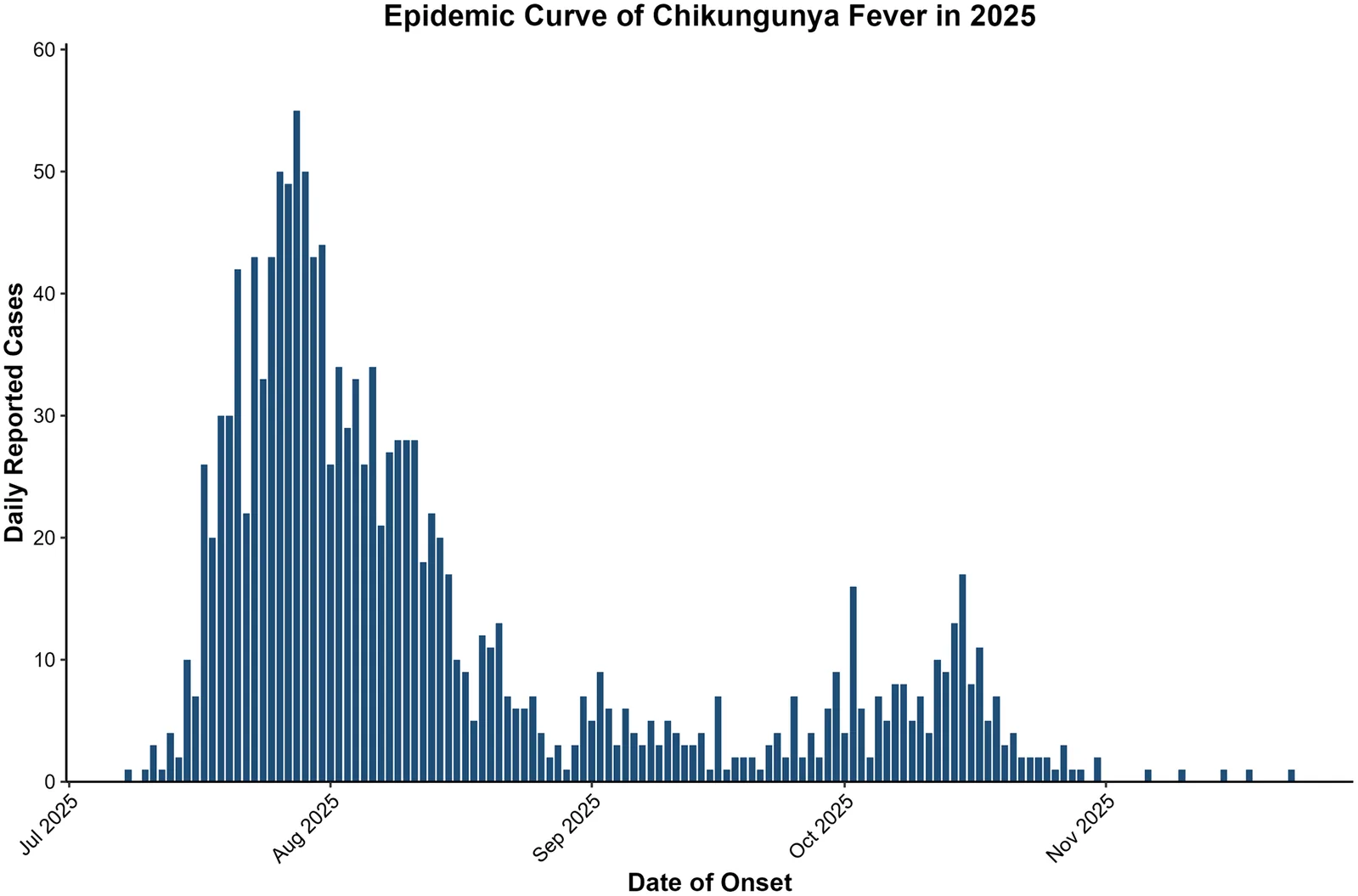
Temporal distribution of locally acquired CHIK cases in Chancheng District, Foshan City, 2025.

The index case of this CHIK outbreak, an imported case, exhibited symptom onset on 8 July, prompting the immediate implementation of comprehensive control measures by local CDC authorities. The epidemic curve demonstrated a unimodal fluctuation, peaking on 28 July with 55 cases reported. Subsequently, case numbers gradually decreased but experienced a brief resurgence in October associated with population mobility during the Mid-Autumn Festival and National Day holidays. The last case exhibited symptom onset on 24 November. Overall, 1,404 locally acquired cases were reported across all 143 villages/communities within the district, with no severe or fatal outcomes recorded. The epidemic curve is presented in Fig 1. Descriptive characteristics of the study variables are summarized in Table 1.

**Table 1:** Descriptive characteristics of village-level variables and Wolbachia intervention status.

Variable	Unit	N	Median (IQR) or n (%)	Minimum-maximum
Cumulative incidence	per 1,000 residents	143	0.81 (0.44-1.55)	0.10-9.97
Population density	10,000 persons/km^2^	143	1.88 (1.01-2.70)	0.14-5.80
Construction-site density	sites/km^2^	143	10.00 (2.00-23.04)	0.00-83.33
Vacant-house density	houses/km^2^	143	45.28 (10.03-134.25)	0.00-1815.38
Rooftop density	rooftops/km^2^	143	333.08 (151.56-706.65)	22.50-3555.56
Narrow-alley density	alleys/km^2^	143	8.14 (0.00-56.67)	0.00-1811.29
Green-space coverage	%	143	2.00 (0.81-8.04)	0.00-58.60
Outdoor mosquito-lamp density	lamps/km^2^	143	3.00 (0.00-38.87)	0.00-1428.57
Hospitalization isolation rate	%	143	82.61 (75.00-100.00)	0.00-100.00
No Wolbachia intervention	villages, n (%)	138	138 (96.5%)	–
Wolbachia intervention	villages, n (%)	5	5 (3.5%)	–

Note. Continuous variables are summarized as median (interquartile range) and minimum-maximum because most village-level variables showed skewed distributions. Wolbachia intervention status is summarized as n (%). The main random forest model included the eight covariates listed as main-model covariates and did not include Wolbachia intervention status.

**Fig 2:**
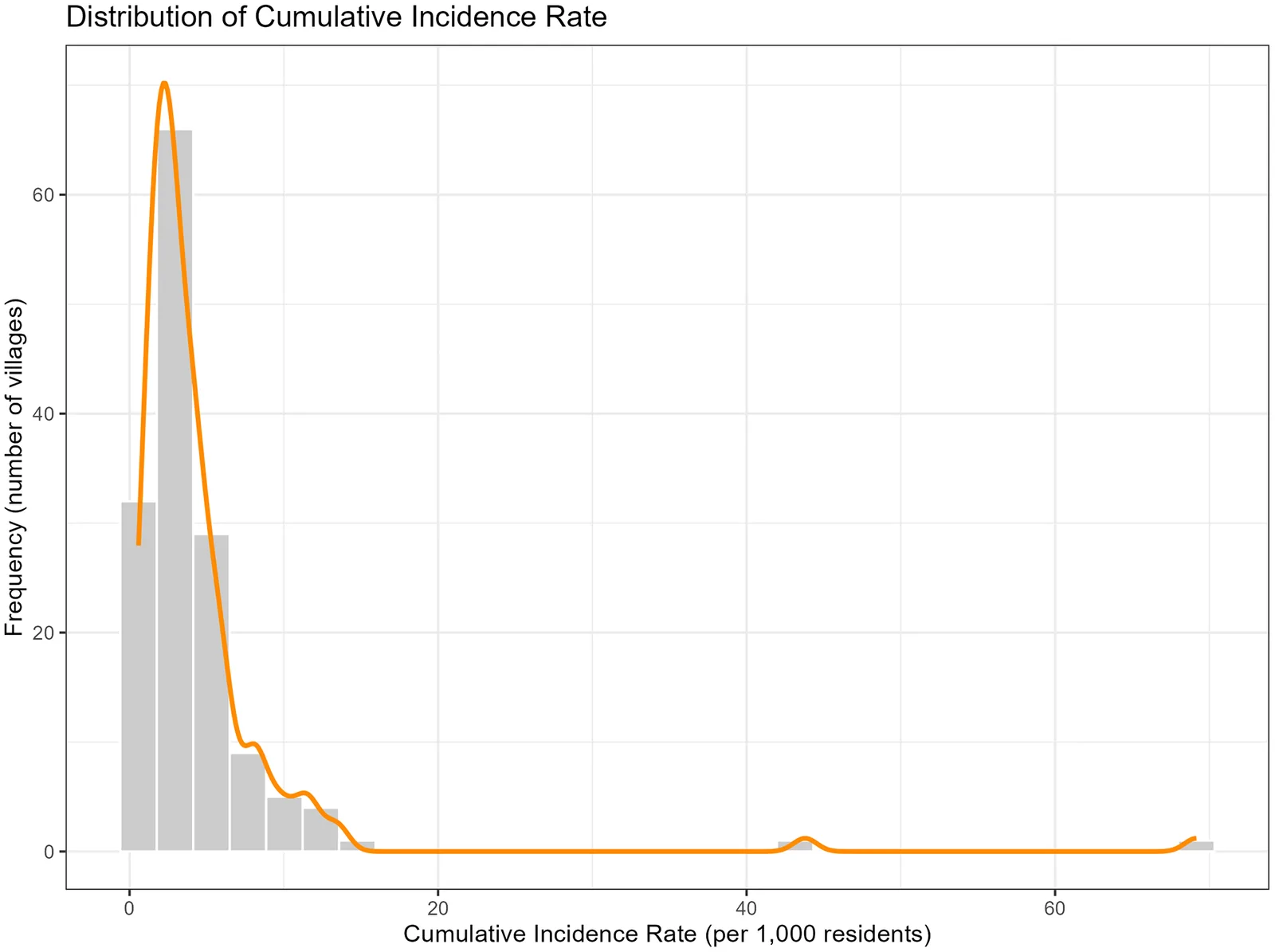
Distribution of cumulative incidence of locally acquired CHIK in Chancheng District, Foshan City, 2025.

**Distribution of incidence across villages/communities:** Among the 143 villages/communities, the median cumulative incidence was 0.81‰ (interquartile range: 0.44‰–1.55‰). All villages/communities reported cases, and the highest recorded incidence reached 9.97‰. The overall distribution was markedly right-skewed (skewness coefficient = 4.00), indicating that the cumulative incidence for most villages/communities was concentrated within the lower range. The cumulative incidence of all villages/communities was below 10‰, and no village/community with an incidence above 10‰ was observed. (Fig 2).

### 3.2. Random forest model construction and interpretation

To elucidate the key environmental and demographic drivers of CHIK incidence in each village/community, this study constructed a random forest model and evaluated its performance based on out-of-bag (OOB) predictions. Variable-importance analysis, 100 repeated modeling runs, SHAP interpretation, and sensitivity analysis were further combined to assess each variable’s contribution to the model’s predictions and its potential nonlinear features.

#### 3.2.1. Model performance evaluation.

Based on OOB validation, the random forest model’s coefficient of determination (OOB R²) on the log(1 + cumulative incidence) scale was 0.206, indicating that the included environmental–social covariates explained approximately 20.6% of the spatial variation in village/community incidence. RMSE (0.387) was higher than MAE (0.286), suggesting that several observations had relatively large prediction errors. After transformation to the original incidence scale, the model’s performance declined (OOB R² = 0.073; RMSE = 1.42‰; MAE = 0.71‰), and the largest positive residuals were concentrated in the quartile group with the highest observed incidence (Q4), confirming that the model systematically underestimated incidence in extremely high-incidence villages/communities. This phenomenon is consistent with the typical features of spatial risk models for vector-borne infectious diseases.

#### 3.2.2. Variable-importance ranking and its stability.

**Fig 3:**
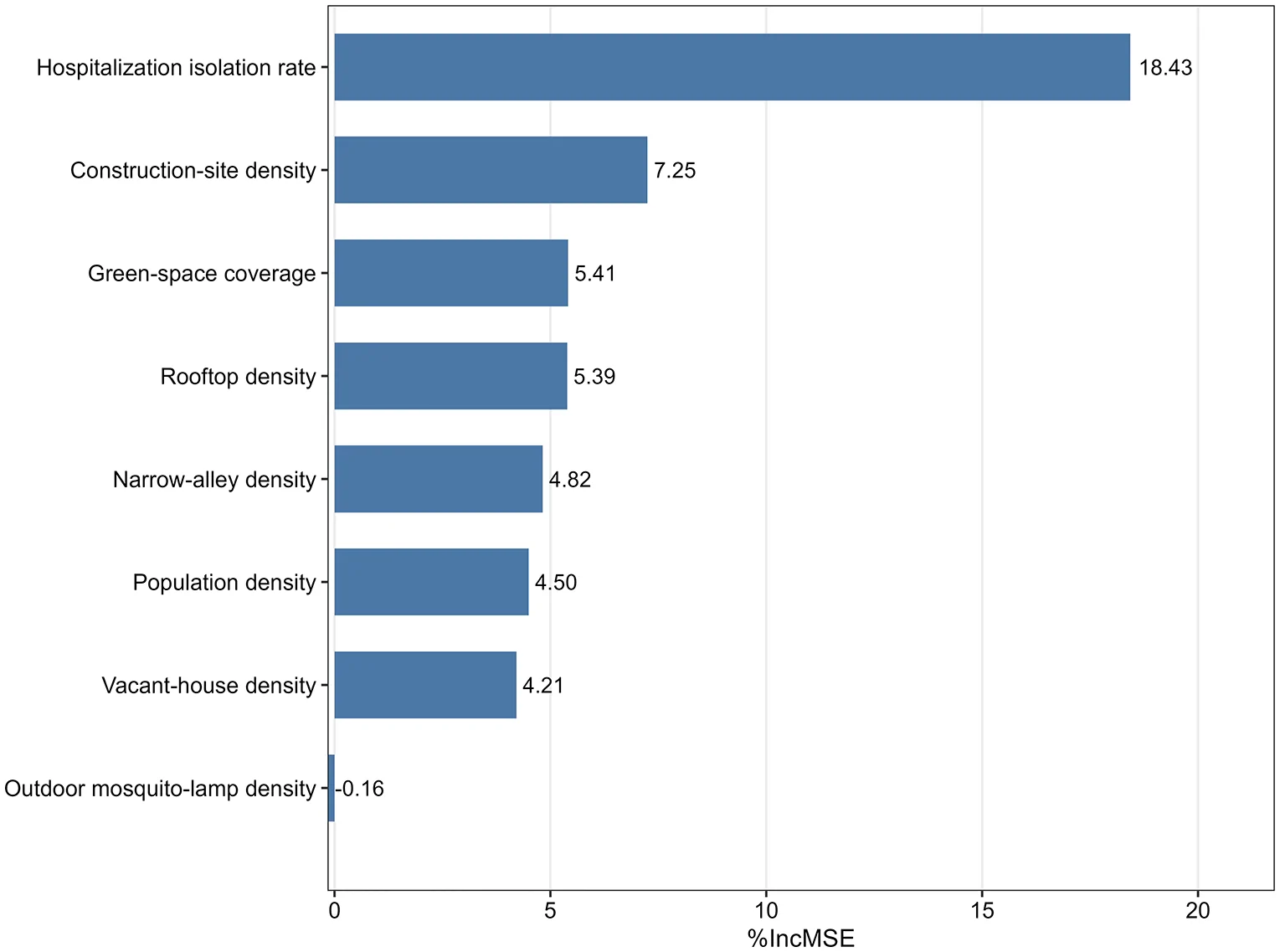
Variable importance in the primary random forest model.

**Fig 4:**
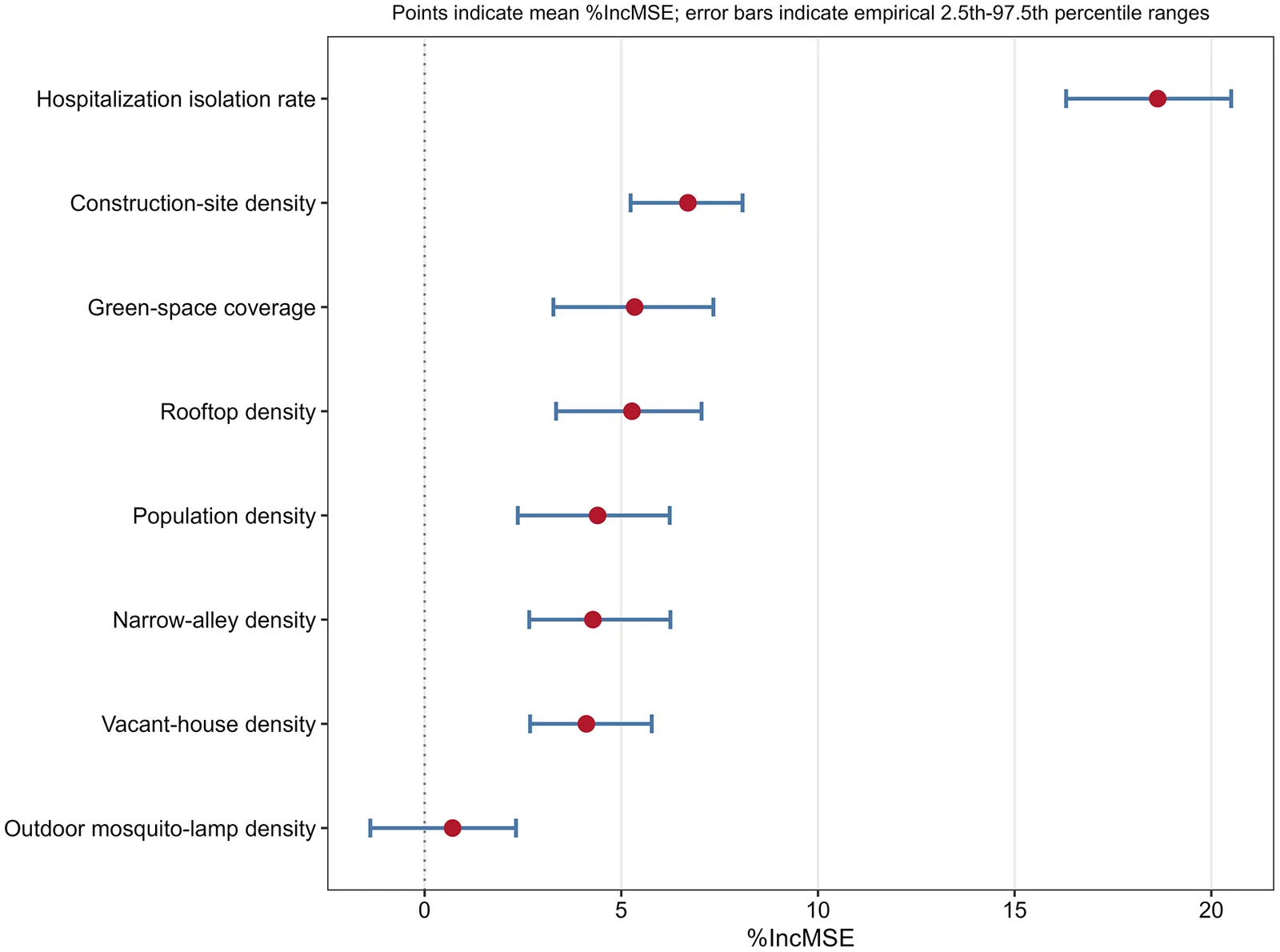
Stability of random forest variable importance.

**Fig 5:**
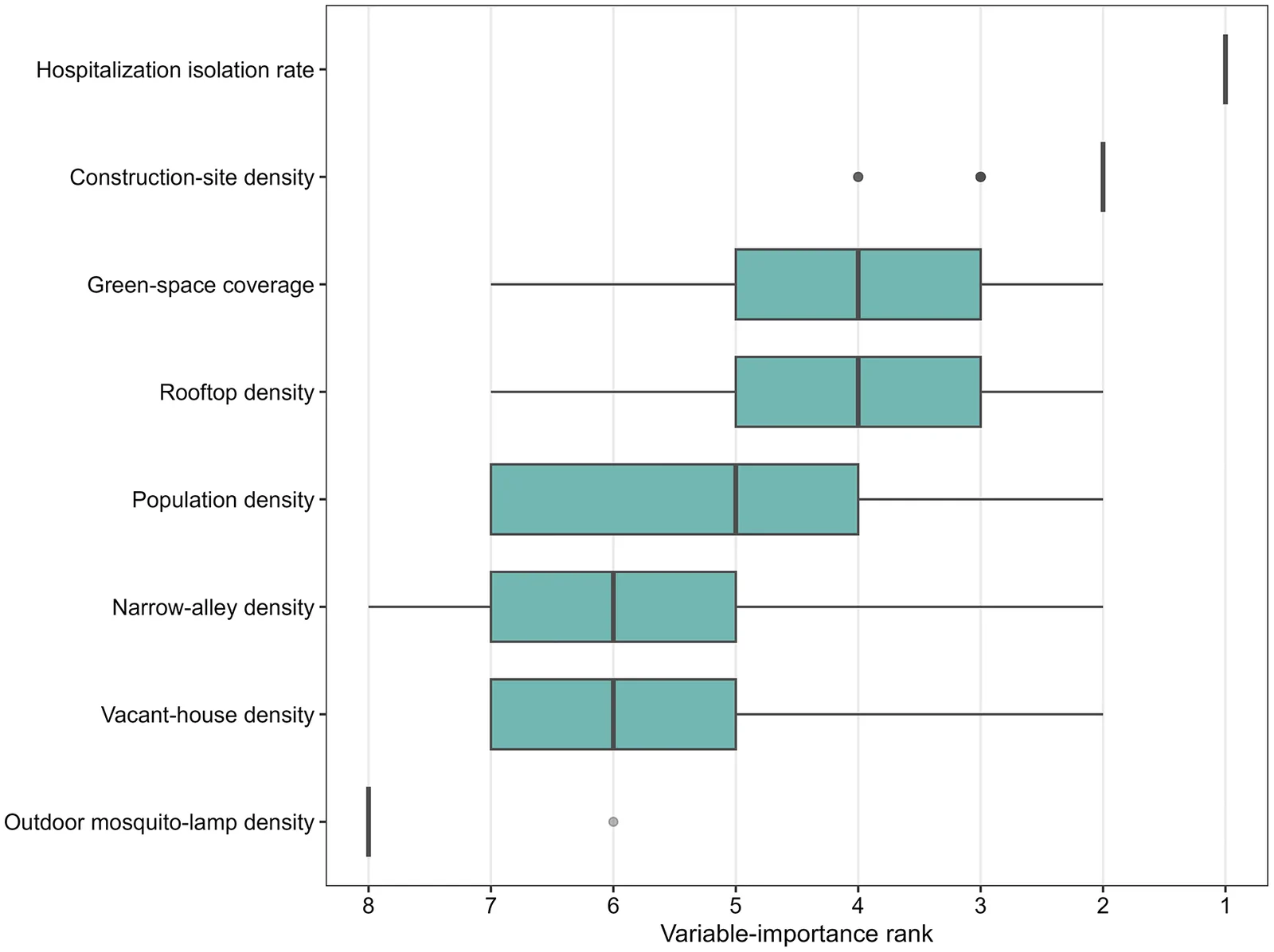
Stability of variable-importance rankings.

The stability test based on 100 repeated modeling runs showed clear differences in each predictor’s contribution to the model (Fig 4). The hospitalization isolation rate showed the highest predictive contribution and was extremely stable, ranking first in 100% of the repeated modeling runs (mean %IncMSE = 18.60; 95% CI: 16.31–20.51). Construction-site density ranked second, with good stability, ranking in the top three in 96% of the repeated modeling runs (mean %IncMSE = 6.69). Green-space coverage and rooftop density had similar importance levels (both with a mean rank of about 4th), but a relatively low proportion of entering the top three (41% and 45%, respectively), indicating that their contribution stability was inferior to that of the aforementioned core factors. In contrast, population density, narrow-alley density, and vacant-house density showed only moderate-to-low predictive contributions, with large fluctuations in ranking. Outdoor mosquito-killing lamp density had the lowest contribution (mean %IncMSE = 0.71; 95% CI: −1.38–2.32), with a top-three proportion of 0%, indicating limited independent predictive value. These results quantified the relative importance and uncertainty of each environmental factor within the predictive framework (Figs 3–5).

#### 3.2.3. Contribution and direction of association of predictors based on SHAP values.

To clarify the degree of contribution and the direction of association of each predictor with the model output, SHAP analysis was performed. The mean absolute SHAP values showed that the hospitalization isolation rate contributed the most to the model’s predictions (mean |SHAP| = 0.102). The Spearman correlation analysis between predictor values and their corresponding SHAP values showed a significant negative correlation (ρ = −0.749, P < 0.001), indicating that, within the model’s feature space, higher isolation rates corresponded to lower predicted incidence. However, given the endogeneity of the hospitalization isolation rate (its value is influenced by both the severity of the epidemic and the intensity of the local control response), this negative association should be strictly interpreted as a predictive-level correlation and should not be inferred as a causal protective effect or an actual intervention effect.

**Fig 6:**
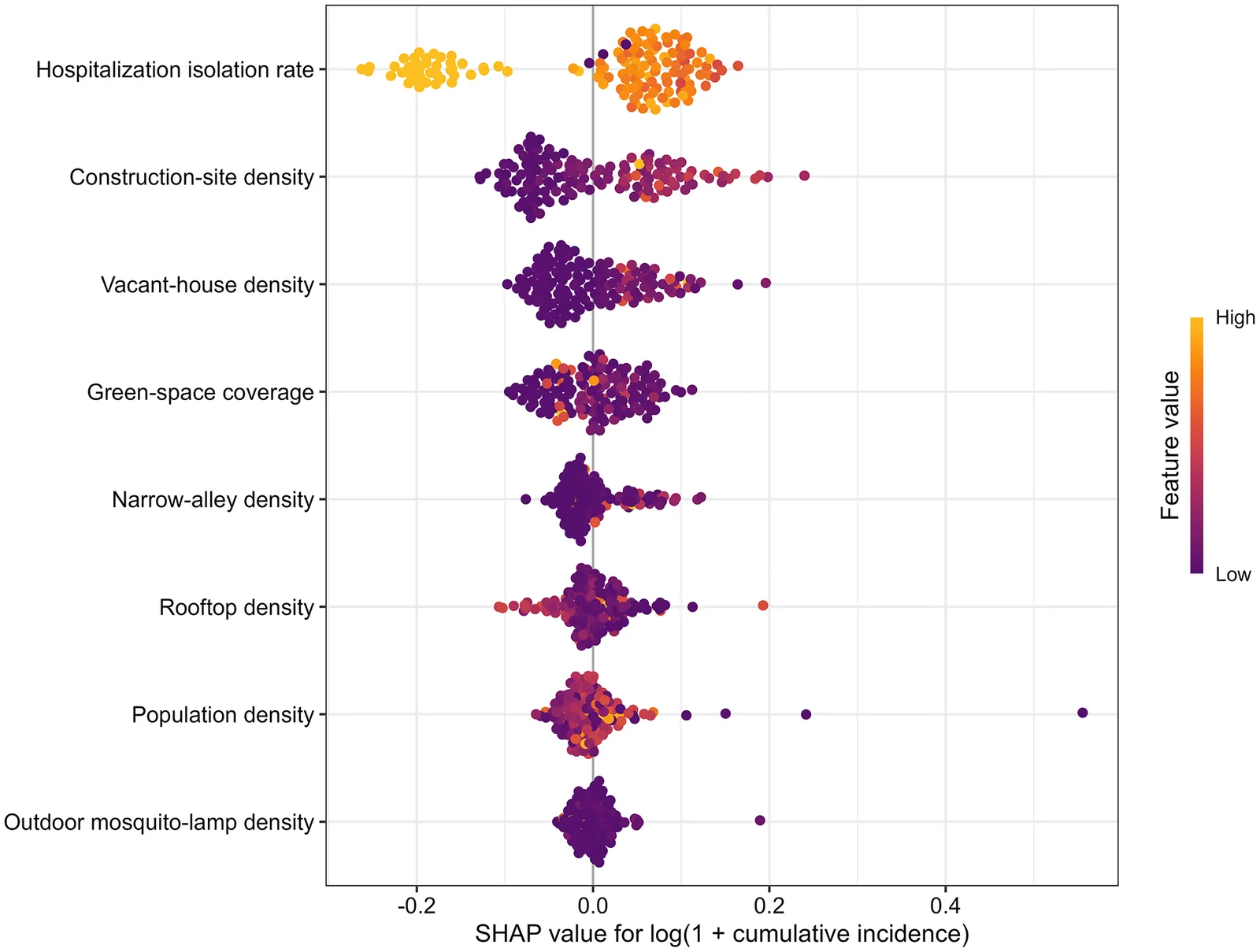
SHAP summary plot for the primary random forest model.

Construction-site density had the next-highest mean absolute SHAP value (mean |SHAP| = 0.071) and was strongly positively correlated with SHAP values (ρ = 0.855, P < 0.001), indicating a stable positive predictive association between construction-site density and the model’s predicted incidence. Similarly, vacant-house density (mean |SHAP| = 0.048, ρ = 0.674, P < 0.001) and narrow-alley density (mean |SHAP| = 0.027, ρ = 0.710, P < 0.001) also showed positive predictive associations. Green-space coverage and population density were positively correlated with SHAP values, but their contribution magnitudes were relatively limited. In contrast, rooftop density (mean |SHAP| = 0.026, ρ = −0.455, P < 0.001) and outdoor mosquito-killing lamp density (mean |SHAP| = 0.015, ρ = −0.372, P < 0.001) were negatively correlated with SHAP values; among these, outdoor mosquito-killing lamp density had the lowest mean contribution magnitude, with a %IncMSE close to zero, indicating a very small independent predictive contribution. In summary, SHAP analysis clarified the directional contribution characteristics of the environmental variables in the model’s predictions, providing a basis for identifying high-risk environmental targets (Table 2 and Fig 6).

**Table 2:** SHAP importance and direction summary for the primary random forest model.

Predictor	%IncMSE	Mean absolute SHAP	Spearman	P value	Direction
Hospitalization isolation rate	18.43	0.102	-0.749	<0.001	Negative
Construction-site density	7.25	0.071	0.855	<0.001	Positive
Green-space coverage	5.41	0.038	0.372	<0.001	Positive
Rooftop density	5.39	0.026	-0.455	<0.001	Negative
Narrow-alley density	4.82	0.027	0.71	<0.001	Positive
Population density	4.5	0.027	0.302	<0.001	Positive
Vacant-house density	4.21	0.048	0.674	<0.001	Positive
Outdoor mosquito-lamp density	-0.16	0.015	-0.372	<0.001	Negative

Note. Mean absolute SHAP represents the average magnitude of each predictor’s contribution to model predictions. Spearman rho was calculated between predictor values and their corresponding SHAP values to summarize the overall direction of association. Positive values indicate that higher predictor values generally increased predicted log(1 + cumulative incidence), whereas negative values indicate that higher predictor values generally decreased predicted log(1 + cumulative incidence). These estimates describe predictive associations and should not be interpreted as causal effects.

#### 3.2.4. Nonlinear associations and threshold features between key predictors and incidence risk.

Based on the SHAP dependence plots, this study further analyzed the nonlinear association features between the main predictors and the model’s predicted incidence. Within this framework, the intersection at which the SHAP value equals zero (SHAP = 0 intersection) was regarded as a potential turning point in the direction of prediction—that is, the point at which a variable’s contribution to the model output changes from negative (decreasing the predicted value) to positive (increasing the predicted value), or vice versa. To assess the stability of such turning points, we used bootstrap resampling (100 times) to construct their empirical distributions and 95% confidence intervals (2.5th–97.5th percentiles).

**Fig 7:**
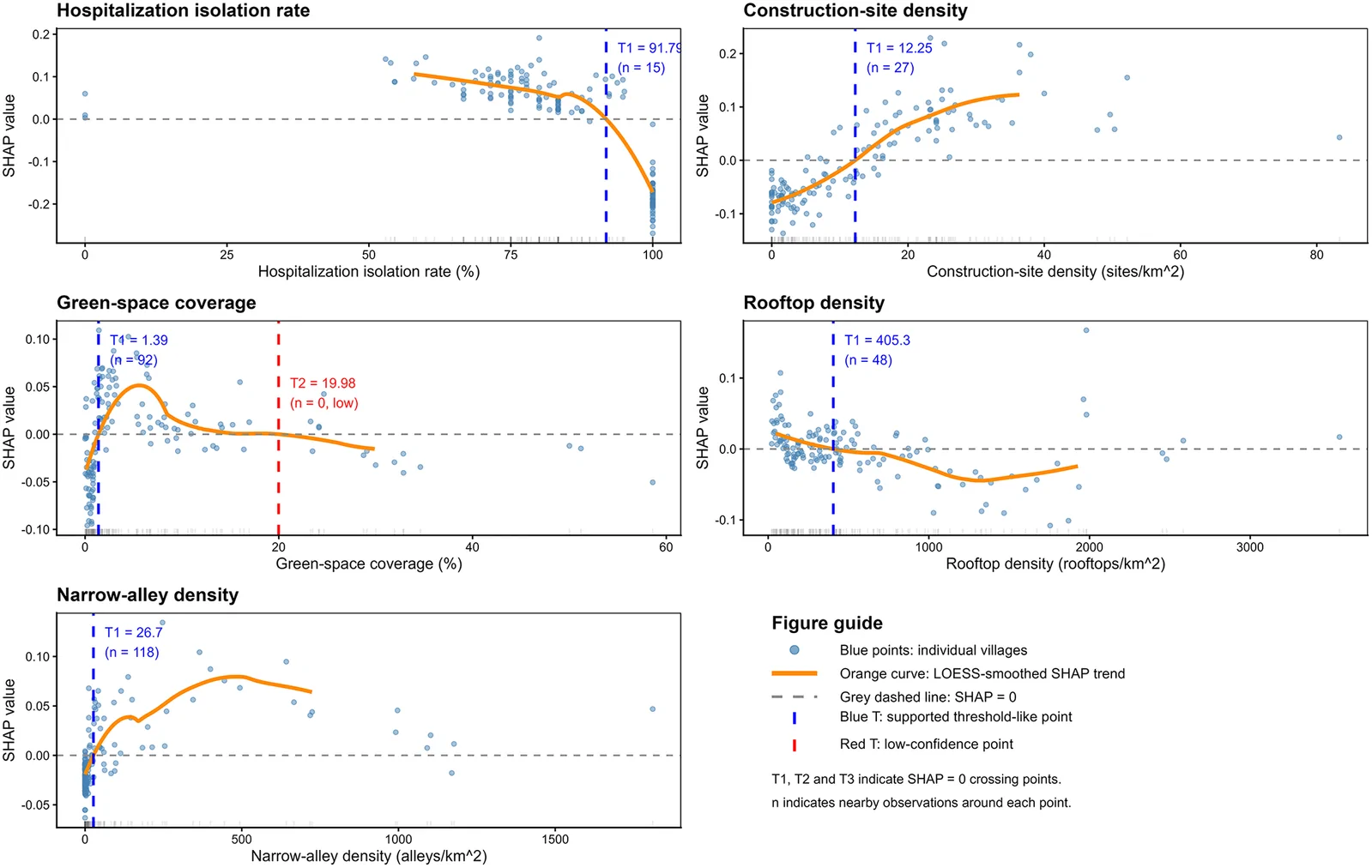
SHAP-derived threshold-like effects of key predictors.

3.2.4.1. Negative predictive association of the hospitalization isolation rate. The hospitalization isolation rate showed a significant negative association with the model’s predicted values. The SHAP = 0 intersection identified in a single main-model run was approximately 91.79%. Bootstrap analysis showed that this turning point was reproduced in 84% of the repeated resamplings, with a median threshold of 92.1% (95% CI: 85.3%–94.6%). Although the mathematical properties of the model indicate that above this threshold a higher isolation rate tends to lower the predicted incidence, given the endogeneity of this variable (confounded by both epidemic severity and control intensity), this turning point reflects only the predictive-level correlation captured by the model and should not be interpreted as a causal protective threshold or an intervention target (Fig 7A).

3.2.4.2. Stable positive threshold-like feature of construction-site density. Construction-site density showed a stable positive nonlinear association with the model’s predicted values. In a single main-model run, the SHAP = 0 intersection was approximately 12.25 sites/km². Bootstrap analysis showed that this turning point was stably identified in 97% of the repeated resamplings, with a median threshold of 12.8 sites/km² (95% CI: 8.48–17.7 sites/km²). Above this level, the contribution of construction-site density to the predicted value changed from negative to positive and showed an increasing trend, indicating that this threshold feature was highly stable and could serve as a potential reference for identifying high-risk environments (Fig 7B).

3.2.4.3. Low-level turning point and non-monotonic feature of green-space coverage. Green-space coverage showed a complex non-monotonic association. A single main-model run identified two SHAP = 0 intersections (1.39% and 19.98%), with relatively adequate sample support near the low-level intersection. Bootstrap analysis showed that the low-level turning point (from negative to positive) was identified in 95% of the resamplings, with a median threshold of 1.53% (95% CI: 0.58%–2.27%), whereas the detection rate of the high-level turning point (from positive to negative) was only 20%. Given the extreme instability and sparse sample support of the high-level turning point, green-space coverage should not be interpreted as an environmental factor with a single stable threshold, and can only be described as having a low-level predictive turning feature (Fig 7C).

3.2.4.4. Non-monotonic and limited-stability threshold pattern of rooftop density. Rooftop density showed a non-monotonic association with the predicted values, with a SHAP = 0 intersection of approximately 405.3 rooftops/km² in a single main-model run. However, the bootstrap results showed large fluctuations in both the location and direction of this turning point, with a detection proportion of 66% and a median threshold of approximately 343 rooftops/km², but with an extremely wide 95% CI (124–932 rooftops/km²) and often accompanied by a secondary intersection in the opposite direction. This indicates that the threshold feature of rooftop density had limited stability and that the turning point identified by the model may be overly influenced by the local data distribution (e.g., villages/communities with extremely high values); its public-health interpretation should be treated with caution (Fig 7D).

3.2.4.5. Threshold-saturation effect of narrow-alley density. Narrow-alley density showed an overall positive predictive association, with a SHAP = 0 intersection of approximately 26.7 alleys/km² identified in a single main-model run. Bootstrap analysis showed that this turning point was identified in 94% of the resamplings, with a median threshold of 27.1 alleys/km², but with a wide 95% CI (8.30–192 alleys/km²). This wide confidence interval indicates that, although the turning point was statistically stable, its exact location was sensitive to the data distribution of high-value villages/communities, and no obvious diminishing marginal effect was observed above the threshold, showing a certain threshold-saturation feature (Fig 7E).

#### 3.2.5. Sensitivity analysis excluding the hospitalization isolation rate.

Given that the hospitalization isolation rate is influenced by the interaction between epidemic severity and control intensity and may carry endogeneity bias, this study constructed a seven-covariate random forest model excluding this variable to assess robustness. After exclusion, the model’s OOB R² decreased from 0.206 to 0.053, and the RMSE increased from 0.385 to 0.423, reflecting the contribution of this variable—as a strong time-dependent factor—to the model fit and also suggesting that the main model may have captured the reverse-causal association introduced by control measures. Nonetheless, the variable-importance ranking changed markedly: construction-site density rose to first place (%IncMSE = 8.54), followed by population density (6.32), narrow-alley density (4.83), and vacant-house density (4.59). The 100 repeated modeling runs further confirmed that construction-site density ranked first in 98% of the iterations and remained in the top three in 100%. In summary, although the hospitalization isolation rate improved the nominal predictive performance, its endogeneity may introduce bias; the sensitivity analysis confirmed that the identification of the core environmental drivers (e.g., construction-site density) was not affected by this interference, and the conclusion is robust.

### 3.3. Model diagnostics and residual analysis

**Fig 8:**
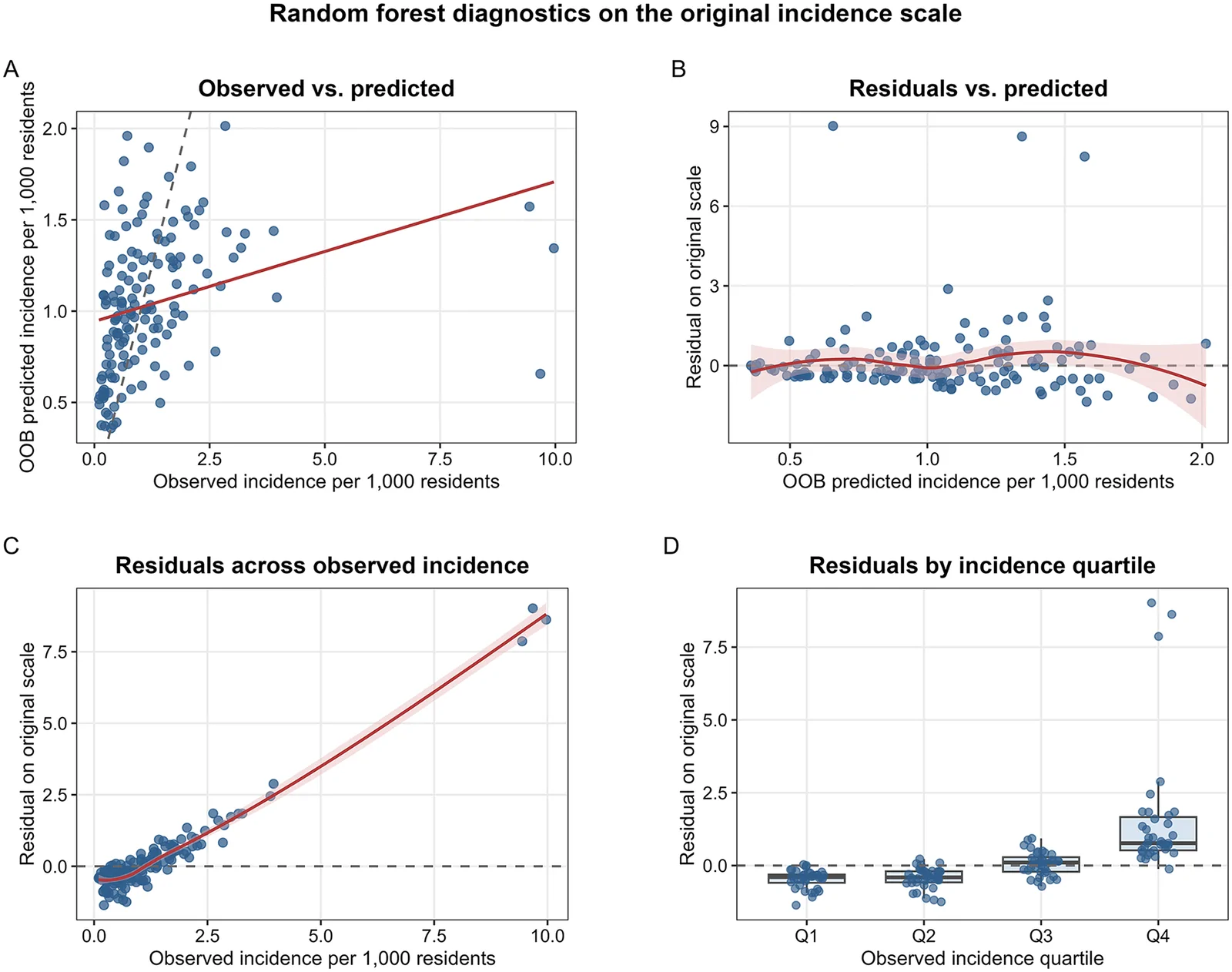
Random forest diagnostics on the original incidence scale.

Model diagnostics showed the random forest model captured part of the overall incidence pattern but showed limited predictive performance, particularly for villages/communities with high incidence. Residual analysis showed that the largest prediction deviations were concentrated in the quartile group with the highest observed incidence (Q4) (Fig 8D), and that this group was dominated by positive residuals. This pattern suggests that the model has limitations in capturing the nonlinear features of extreme transmission intensity, which may lead to conservative estimates of risk in high-risk villages/communities.

## 4. Discussion

This study jointly applied random forest regression and the SHAP interpretability algorithm to systematically analyze the drivers of village/community-level CHIK incidence during the 2025 outbreak in Chancheng District, Foshan City. Descriptive analysis showed that the outbreak had a typical right-skewed distribution (skewness = 4.00), with a median cumulative incidence of 0.81‰ and a maximum of 9.97‰, and that all villages/communities reported cases. Against this background, the model identified the hospitalization isolation rate and construction-site density as the two factors contributing most prominently to the model’s predictions. However, it must be emphasized that this study aimed to identify predictive associations to assist risk stratification, rather than to establish causal effects.

The hospitalization isolation rate contributed most prominently to the model’s predictions and was significantly negatively correlated with the SHAP values. This observational association is directionally consistent with the assessment by Wu et al. based on a dynamic model, which confirmed that increasing the case isolation rate can effectively reduce the outbreak size by interrupting transmission [34]. However, there is a fundamental methodological difference between the theoretical expectations of mechanistic models and the statistical inference from observational data. The negative association identified in this study is very likely driven by reverse causation—that is, the passive rise in isolation rates was caused by the intensified control measures triggered by high incidence, rather than by the control measures themselves. This indicates that what the model captured is a time-lagged correlation between the “control response” and the “epidemic burden,” rather than a causal protective effect of isolation measures. The sensitivity analysis further confirmed that, after removing this endogenous variable, although the model fit decreased (OOB R²: 0.206 → 0.053), the ranking of environmental factors centered on construction-site density remained robust (ranking first in 98% of repeated modeling runs). Therefore, the related thresholds are of statistical descriptive significance only and should not be extrapolated as intervention targets; this highlights the inherent limitation of observational data in examining predictive associations involving control measures.

Construction-site density was the most stable environmental predictor in this study. SHAP analysis showed that it was strongly positively correlated with the predicted values (ρ = 0.855, P < 0.001) and had a stable positive threshold feature at approximately 12.8 sites/km² (bootstrap 95% CI: 8.48–17.7 sites/km²). This result is consistent with common knowledge in mosquito-borne ecology: construction sites, owing to the presence of numerous standing-water containers (e.g., sedimentation pits, foundation pits), are core breeding sites for Aedes mosquitoes [35], and various containers and materials are also important vehicles for the dispersal of vector mosquitoes [36]. Notably, only 5 villages/communities (3.50%) in the study area implemented biological control such as “mosquitoes against mosquitoes,” which to some extent rules out interference from novel interventions with the construction-site density risk signal and strengthens the explanatory power of this environmental factor as a core predictor. This provides a potential quantitative basis for designating such sites as “priority control areas” and implementing stratified interventions (e.g., intensifying inspection and clean-up frequency in high-density areas), and also corroborates the effectiveness of China’s dengue control experience centered on environmental management [37].

This study further revealed a paradigm shift in disease drivers in high-density built environments. Although rooftop density and green-space coverage showed nonlinear associations, their independent predictive contributions were weak and their SHAP thresholds were highly unstable (e.g., the bootstrap detection rate for rooftop density was only 66%, 95% CI: 124–932 rooftops/km²), making them difficult to use as robust targets; although narrow-alley density and vacant-house density showed stable positive associations, their importance was moderate-to-low and their marginal contributions were limited. Notably, despite the dense population of Chancheng District, the predictive role of population density was likewise weak and lacked a clear threshold, in sharp contrast to previous conclusions emphasizing it as a core predictor of mosquito-borne diseases [34,38–40]. On this basis, this study supports the inference of Ren et al. (2019) [41] that, in highly urbanized core areas, the risk drivers may have shifted from the macro-level “scale of population aggregation” to the micro-level “frequency of contact between people and high-risk environments (e.g., construction sites).” The fine-scale distribution of breeding or resting habitat types may better explain micro-level risk heterogeneity than macro-level population indicators. However, the cross-sectional exposure assessment used in this study is unable to capture the dynamic evolution of environmental factors and their time-varying association with incidence risk. Future research urgently needs to adopt a time-matched longitudinal design to accurately quantify the differential driving effects of environmental factors at different stages of the outbreak, and thereby build more timely dynamic early-warning models.

### 4.1. Comparison with existing studies

Compared with published studies of the 2025 Foshan CHIK outbreak, this study is fundamentally different in both methodology and objectives. Existing literature has mostly focused on transmission dynamics and clinical characterization: for example, Zhang et al. [12] and Zhao et al. [42] quantified the very high basic reproduction number (R₀) through statistical inference and an SEIR model, respectively, aiming to analyze the theoretical transmission intensity of the outbreak and the “human–mosquito” transmission mechanism; in addition, Lv et al. and Li et al. [43,44] reported the spatial distribution characteristics of the outbreak and the field response, while Wang et al. [45] focused on the age-stratified clinical presentation and virological characteristics of patients. Although these studies deepened understanding of the virus’s transmission potential, they mostly relied on pre-specified parameters or qualitative descriptions and struggled to characterize predictive associations involving complex control measures.

In contrast, this study shifted the focus from characterizing transmission dynamics to identifying community-level predictive patterns. This study is the first to introduce an interpretable machine-learning framework in this outbreak, breaking through the linear assumptions and pre-specification limitations of traditional models and directly inverting the complex associations between environmental variables and incidence risk from high-dimensional observational data. Through the SHAP framework, this study not only identified key predictors but also revealed their nonlinear threshold features (e.g., the stable turning point of construction-site density), advancing risk assessment from macro-level parameter estimation to quantitative early warning in a micro-level feature space. This empirically based association analysis provides a scientific basis for building a data-driven, zoned and stratified precision control system, and compensates for the shortcomings of traditional dynamic models in examining predictive associations involving complex real-world interventions.

### 4.2. Study limitations

This study has several limitations that should be considered when interpreting the results.

First, inherent constraints of a cross-sectional ecological design. Because the analysis used villages/communities as the unit of measurement, associations identified herein represent group-level statistical relationships rather than causal relationships at the individual level. SHAP values quantify variables’ predictive contributions to the model’s predictions, not causal effects.

Second, there were limitations related to data and variable selection. The model accounted for approximately 20.6% of the spatial variation in CHIK incidence, indicating that some influential factors were not captured. This limitation primarily stems from two aspects. First, as the study was conducted within the same administrative district (Chancheng District), where meteorological conditions such as temperature, humidity, and precipitation are relatively homogeneous, these factors could not be included as explanatory variables in the study design. Additionally, mosquito vector density data obtained from emergency surveillance in 2025 were not incorporated into the analysis due to the overall risk level remaining consistently low. The absence of these key environmental and vector dynamic variables may have omitted some important transmission drivers, thereby limiting the model’s overall explanatory power. Additionally, measurements of certain environmental variables (e.g., construction sites and green-space coverage) excluded areas managed at or above the town/subdistrict level, potentially introducing measurement errors. Furthermore, the “mosquitoes against mosquitoes” intervention involved only five pilot villages/communities, leading to a limited sample size that may have underestimated its true effect. Nonetheless, under existing data constraints, this study identified multiple key factors with clear predictive associations and their thresholds through a random forest model, providing an evidence-based reference for prioritizing on-site interventions. Future research should further validate and expand upon these findings with more comprehensive data.

Third, temporal mismatch. This study has an inherent temporal-mismatch limitation: the outcome variable (cumulative incidence) integrates the cumulative risk over the entire outbreak period, whereas the core environmental predictors (e.g., construction-site and vacant-house density) were based only on a single-time-point measurement in August 2025. Although these time-point data effectively captured the environmental pressure during the outbreak peak, their heterogeneity relative to the environmental conditions in the early stage of the outbreak may cause the model to underestimate the role of early drivers, and it is also difficult to clarify the causal temporal sequence between environmental evolution and outbreak progression. Future research urgently needs to adopt a time-matched longitudinal design, using multi-time-point dynamic monitoring of environmental variables to accurately analyze their dynamic evolution and time-varying association with incidence risk.

Fourth, the limited model applicability to extremely high-incidence scenarios. The model’s fitting performance declined for samples with higher incidence (>10‰), suggesting diminished capability to explain and predict severe outbreaks. In high-intensity transmission contexts, outbreak progression may increasingly depend on unmeasured complex social factors, such as large-scale population movements and altered community behaviors, that static or semi-static variables used in this analysis could not adequately capture.

Fifth, absence of spatial validation. Limited by the inability to obtain complete high-resolution spatial boundary data for all 143 villages/communities, this study did not perform spatial autocorrelation tests (e.g., Moran’s I) or spatial block cross-validation analysis. Future research should supplement this with complete GIS data to rule out potential interference from spatial dependence on the model results.

Sixth, the spatiotemporal extrapolation of the conclusions requires cautious validation. Findings from this study reflect the specific conditions of a high-intensity outbreak within a particular urban setting. Consequently, identified key risk factors and thresholds (e.g., construction-site density threshold of 12.8 sites/km²) may be strongly influenced by local socioeconomic and environmental contexts. Extrapolating these results to other regions, differing transmission intensities, or alternative urban structures requires cautious validation and calibration with local data. Future research should integrate dynamic data at higher spatiotemporal resolutions and validate the model across varied epidemiological settings to enhance its generalizability and practical utility for public health decision-making.

### 4.3. Policy implications and future research directions

Despite the limitations noted above, this study provides valuable preliminary evidence derived from real-world data and interpretable machine-learning methods, contributing to precision-based control practices and informing future research on mosquito-borne infectious diseases.

#### 4.3.1. Policy implications: Transitioning from “experience-driven” to “data-driven” precision risk stratification.

4.3.1.1. Building a stratified response mechanism based on environmental thresholds. This study found that construction-site density (bootstrap median 12.8 sites/km²) showed a relatively stable threshold-like pattern. This suggests that, during the early stage of an outbreak or in routine monitoring, GIS technology can be used to map “high-risk breeding-site heat maps,” using the above thresholds as early-warning lines for triggering intensified inspection and clean-up. For areas exceeding the thresholds, higher-frequency vector surveillance and more intensive environmental remediation can be automatically triggered, achieving precise allocation of control resources and avoiding a “one-size-fits-all” approach.

4.3.1.2. Establishing a priority-control sequence for different types of breeding or resting sites. Variable-importance analysis clearly showed that construction sites and vacant houses are environmental factors with predictive contributions, and their importance is significantly higher than that of factors such as rooftops and green-space coverage. This provides a scientific ranking for allocating limited control manpower and resources: inspection and management should prioritize construction sites and vacant houses, especially points with unclear ownership and absent management, rather than distributing efforts evenly across all potential breeding or resting sites.

4.3.1.3. Cautiously interpreting the early-warning value of the hospitalization isolation rate. Although the sensitivity analysis suggested that the hospitalization isolation rate carries significant endogeneity bias (reverse causation), its strong predictive contribution in the model still has reference value. The hospitalization isolation rate may serve as a descriptive indicator of the intensity of the public health response and healthcare-resource use; however, its temporal early-warning value requires validation using longitudinal data. However, caution is needed: the threshold identified by the model (e.g., 92%) should not be directly used as an intervention target, to avoid falling into the ecological fallacy.

4.3.1.4. Reshaping the risk-assessment paradigm for high-density urban areas. This study found that in highly urbanized central urban districts such as Chancheng, the independent predictive role of population density is weak. This suggests that, in micro-level built environments, the risk drivers may have shifted from the macro-level “population aggregation” to the micro-level “frequency of contact between people and high-risk environments.” Therefore, future risk assessment should go beyond general models that rely solely on population density and instead adopt refined models that integrate specific environmental features (e.g., construction-site density, narrow-alley density) to capture micro-level risk heterogeneity.

#### 4.3.2. Future research directions.

4.3.2.1. Data integration and model validation: Future research should prioritize the integration of dynamic, high-resolution, multi-source data, including daily meteorological variables, real-time vector density indicators (e.g., the Breteau Index derived from ovitraps), population mobility data from mobile-phone signaling, and more comprehensive socioeconomic and building-related datasets. On this basis, the generalizability and stability of the risk factors and thresholds identified in this study should be validated across outbreaks in diverse geographic settings and varying transmission intensities, and predictive models incorporating dynamic features should be developed.

4.3.2.2. Strengthening causal inference, mechanistic validation, and temporal matching. This study has an inherent temporal mismatch—cumulative incidence reflects whole-period risk, whereas the core environmental factors (e.g., construction-site and vacant-house density) were measured at only a single time point in August 2025, making it difficult to clarify the causal temporal sequence and prone to underestimating early drivers. Future research urgently needs to adopt a time-matched longitudinal design, using multi-time-point dynamic monitoring to analyze the time-varying association between environmental evolution and incidence risk and to correct the estimation bias of single-time-point measurement. On this basis, quasi-experimental designs (e.g., interrupted time series, natural experiments) can be combined to disentangle the net effects of measures such as “mosquitoes against mosquitoes” and isolation control. At the same time, the potential mechanisms of nonlinear relationships involving narrow alleys and rooftops need to be explored in depth: at the biological level, focusing on the vector-carrying capacity of specific standing-water containers, and at the social level, focusing on community participation and the effectiveness of grid-based management, so as to build a complete evidence chain from statistical prediction to biological causation.

4.3.2.3. Optimizing model structure and expanding validation. In response to the limitations of the model’s limited explanatory power in high-intensity outbreaks and the absence of spatial validation, future research should build hybrid models incorporating spatiotemporal random effects to capture the complex driving mechanisms under extreme transmission. Given that this study was limited by single-time-point measurement, subsequent modeling should incorporate multi-time-point dynamic data to improve whole-period fitting capability. At the same time, complete GIS boundaries should be used to conduct spatial block cross-validation, and key thresholds (e.g., construction-site density of 12.8 sites/km²) should be externally calibrated across different urban forms to establish the model’s robustness and extrapolation applicability.

## 5. Conclusions

Based on data from 1,404 local CHIK cases across the 143 villages/communities of Chancheng District, Foshan City, this study constructed a random forest regression model combined with the SHAP interpretability algorithm. The results showed that the hospitalization isolation rate and construction-site density were identified as the most stable core predictors, whereas the traditionally emphasized narrow-alley density, vacant-house density, and macro-level population density had limited contributions and fluctuating rankings. The results reveal that, in high-density urban built environments, the drivers of disease risk have shifted from macro-level population aggregation to the coupling of micro-level environmental exposure and control response. It must be emphasized that the associations identified in this study are predictive; limited by endogeneity and the cross-sectional design, the related thresholds should not be directly extrapolated as causal targets, and their robustness remains to be further validated with time-matched longitudinal data.

## Supporting information

S1 Data Village/community-level chikungunya incidence and associated-factor data from Chancheng District, Foshan City. The dataset contains the cumulative incidence of chikungunya in 143 villages/communities in Chancheng District, Foshan City, together with nine candidate predictors: population density, construction-site density, vacant-house density, rooftop density, longitudinal narrow-alley density, green-space coverage, number of outdoor mosquito-killing lamps, implementation of the Wolbachia-based mosquito-control intervention, and hospitalization isolation rate.
(XLSX)
